# Naive skepticism scale: development and validation tests applied to the chilean population

**DOI:** 10.1186/s41155-024-00288-0

**Published:** 2024-02-20

**Authors:** Rodrigo Ferrer-Urbina, Yasna Ramírez, Patricio Mena-Chamorro, Marcos Carmona-Halty, Geraldy Sepúlveda-Páez

**Affiliations:** Escuela de Psicología y Filosofía, Universidad de Tarapacá, Arica, Chile

**Keywords:** Naive Skepticism, Measurement, Misinformation, Scale development

## Abstract

**Background:**

Skepticism has traditionally been associated with critical thinking. However, philosophy has proposed a particular type of skepticism, termed naive skepticism, which may increase susceptibility to misinformation, especially when contrasting information from official sources. While some scales propose to measure skepticism, they are scarce and only measure specific topics; thus, new instruments are needed to assess this construct.

**Objective:**

This study aimed to develop a scale to measure naive skepticism in the adult population.

**Method:**

The study involved 446 individuals from the adult population. Subjects were randomly selected for either the pilot study (phase 2; *n* = 126) or the validity-testing study (phase 3; *n* = 320). Parallel analyses and exploratory structural equation modelling were conducted to assess the internal structure of the test. Scale reliability was estimated using Cronbach's alpha and McDonald's omega coefficients Finally, a multigroup confirmatory factor analysis was performed to assess invariance, and a Set- Exploratory Structural Equation Modeling was applied to estimate evidence of validity based on associations with other variables.

**Results:**

The naive skepticism scale provided adequate levels of reliability (ω > 0.8), evidence of validity based on the internal structure of the test (CFI = 0.966; TLI = 0.951; RMSEA = 0.079), gender invariance, and a moderate inverse effect on attitudes towards COVID-19 vaccines.

**Conclusions:**

The newly developed naive skepticism scale showed acceptable psychometric properties in an adult population, thus enabling the assessment of naive skepticism in similar demographics. This paper discusses the implications for the theoretical construct and possible limitations of the scale.

In recent literature, misinformation is understood as any partially false information (Ecker et al., 2022; Wang et al., 2019), with focus on its consequences, such as influencing the spread of risky health behaviors (Wang et al., 2019). Misinformation is identified as the leading direct cause of death among young people and an indirect cause in adults (WHO, 2020). Its impact was particularly evident during the COVID-19 pandemic, notably affecting attitudes towards vaccines (Dubé et al., 2022; Zheng et al., 2022). These attitudes can be influenced by a lack of knowledge or a predisposition to assimilate false or biased information, thus increasing misbeliefs about the consequences of risky health behaviors. Therefore, a plausible hypothesis is that naive skepticism affected attitudes towards vaccines in the context of COVID-19 (Bavel et al., 2020; Roozenbeek & van der Linden, 2019; Roozenbeek et al., 2020). To this end, part of the scientific community is investigating variables that heighten susceptibility to misinformation, suggesting various psychological traits as protective (e.g., analytical thinking, deductive and inductive reasoning) (Sinderman et al., 2020) or risk factors (e.g., receptivity to nonsense, political orientation, religious beliefs) (Gligorić et al., 2022; Pennycook & Rand, 2019a).

From the perspective of educational philosophy, the concept of naive skepticism has emerged. It potentially impacts the ability of young adults to correctly judge the veracity of information (Wright, 2019), thus playing a role in discriminating between false and accurate information. Naive skepticism is defined as a psychological trait characterized by the tendency to dismiss information without critical analysis, even when such information is supported by truthful, or at least reasonably acceptable, evidence. This trait is distinct from reasoned skepticism, which is based on arguments or evidence supporting the skeptical stance (Wright, 2019). Furthermore, naive skepticism differs from conspiracy theories. Naive skepticism corresponds to a general tendency to question the credibility of official sources, whereas conspiracy theories are associated with persons who believes in specific conspiracies or have a strong inclination towards conspiracy thinking; often linked to varied topics, making it less applicable in different contexts (Douglas et al., 2019; Imhoff & Lamberty, 2018; Van Prooijen & Douglas, 2017; Quiring et al., 2021). Notably, naive skeptics are predisposed to accepting conspiracy theories (Quiring et al., 2021).

Regarding how naive skepticism might increase vulnerability to disinformation, it has been suggested that naive skepticism influences the level of information processing, primarily through reasoning motivated by maintaining one's beliefs (Wood & Porter, 2019). This reasoning makes individuals more susceptible to disinformation that aligns with their central belief systems or group identity (Erion, 2005). Consequently, naive skepticism emerges as a risk factor, leading to a higher tendency to believe in disinformation (Wright, 2020).

Naive skepticism usually manifests as a reluctance to trust official information sources, which, in the context of healthy democracies, typically base their communications on evidence or reasonable conjecture. Notable sources often subjected to unfounded questioning by individuals with higher levels of naive skepticism include scientific organizations (e.g., Steffens et al., 2019), governmental organizations (e.g., Van Scoy et al., 2021; Lynch, 2023), and mainstream media (e.g., Nekmat, 2020).

Given its explanatory and predictive potential regarding susceptibility to misinformation, some scales have been developed to assess skepticism as a naive trait. These include the following: 1) Skepticism Towards Advertising Scale, which measures skepticism towards advertisements using 9 items (e.g., “We can depend on getting the truth in most advertising”) and has a unidimensional structure (Obermiller & Spangenberg, 1998); 2) Climate Change Skepticism Scale, assessing skepticism towards climate change through 3 items (e.g., “I doubt that there is global warming going on”) (Ojala, 2015); and, 3) Professional Skepticism Scale, measuring professional skepticism in the audit process multidimensionally (i.e., as an individual trait and as a state in professionals) with 30 items (e.g., “I often accept other people's explanations without further thought”) (Hurtt, 2010). However, these scales are domain-specific and do not assess naive skepticism as a general trait, as conceptualized by Wright (2020).

Despite the relevance of naive skepticism as a general trait in susceptibility to misinformation, empirical studies supporting this notion are limited. This paucity may be due to the lack of measurement scales that provide valid and reliable means of testing this and other related hypotheses. Consequently, the aim of this study was to develop a new scale to measure naive skepticism, providing evidence of its validity, reliability, and invariance in an adult population. Given the nascent nature of this field of study and the scarcity of prior research, establishing equivalence between men and women in comprehending the concept of naive skepticism was crucial. This aligns with the ongoing debate surrounding the gender similarity hypothesis, which considers the potential for theorizing similarities between men and women (Hyde, 2014). Hence, gender invariance was a focus. This new scale will expand the capabilities for studying susceptibility to misinformation and its ramifications.

## Materials and method

### Participants

This research, an instrumental study with a cross-sectional design (Ato et al., 2013), was conducted in three primary phases. Phase 1 involved drafting new items, which were then evaluated by expert judges (refer to the *Instruments* section for more details). Phase 2 focused on exploring the dimensionality of the scale (i.e., the revised, post expert-review version). The final phase involved assessing scale validity based on the internal structure of the test and its associations with other variables. It is important to note that only the last two phases had distinct data sets.

In Phase 2, a total of 126 adults participated. Of these, 55.6% (*n* = 70) were female and 44.4% (*n* = 56) were male, with a mean age of 24 years (SD = 7.01). Phase 3 saw the participation of 320 adults, with 54.4% (*n* = 174) female, 44.7% (*n* = 143) male, and 0.6% (*n* = 2) identifying as non-binary. The mean age in this phase was 29.32 years (SD = 11.01). Detailed demographic information for Phase 3 is presented in Table 1.

**Table 1:** Sociodemographic characteristics of the study (Phase 3)

		M (SD) or N (%)
Gender	Male	143 (44.7%)
	Female	174 (54.7%)
	Non-binary	2 (0.6%)
	Not reported	1 (0.3%)
Age (years)		29.32 (11.01)
Study Areas	Health Sciences	65 (20.3%)
	Engineering	57 (17.8%)
	Social Sciences	116 (36.3%)
	Education	25 (7.8%)
	Business and Administration	14 (4.4%)
	Arts and Architecture	6 (1.9%)
	Basic Sciences	5 (1.6%)
	Agricultural Sciences	2 (0.6%)
	Not reported	30 (9.4%)
Schooling level	University Education Complete	103 (32.2%)
	Incomplete University Education	146 (45.6%)
	Technical Education Complete	30 (9.4%)
	Secondary Education Complete	28 (8.8%)
	Technical Education Incomplete	8 (2.5%)
	Elementary Education	4 (1.3%)
	Incomplete Secondary Education	1 (0.3%)

*M* = Mean, *SD* Standard deviation, *N* Number of subjects, % = Percentage

### Instruments

*Naive Skepticism Scale (NSS)*: The NSS is an ad-hoc instrument designed to assess the level of naive skepticism in individuals. It comprises two dimensions: skepticism towards governmental organizations and the official press (hereafter referred to as SGO; 7 items) and skepticism towards science (hereafter referred to as SS; 7 items). The scale utilizes a Likert format with five response categories, ranging from 1 = "Never" to 5 = "Always." The items in the NSS are statements reflecting distrust towards science, governmental organizations, and the press.

Given the absence of standardized instruments for measuring naive skepticism broadly, the initial Phase involved developing a comprehensive operational definition based on a literature review. This definition encompassed the overall tendency to reject information without critical analysis or the support of reliable evidence, as well as specific aspects for each sub-dimension (i.e., SGO, SS). Subsequently, 37 items were drafted following the guidelines for creating Likert-type scale items, as proposed by AERA, APA & NCME (2014) and Muñiz and Fonseca-Pedrero (2019). These items were then evaluated by four expert judges (all from the social sciences field; three doctoral researchers and one master's student) for grammatical adequacy (coherence and clarity) construct representativeness. Judges individually scored each item on a scale of 1, 0, and -1, where "1" indicated grammatical adequacy and construct representativeness. Items with means less than or equal to 0 were discarded.

The revised version resulted in a 23-item scale, which was used in Phase 2 (*n* = 126) for initial dimensionality exploration. Item selection was guided by content relevance, corrected homogeneity index, and parallel analysis. The process culminated in a debugged 14-item scale used in Phase 3 in the adult population. Detailed psychometric evidence for this final version is presented in the *Results* section.

*Spanish Version of the COVID-19 Vaccine Attitude Scale (*Campo-Arias et al., 2021*)*: This 8-item instrument measures favorable attitudes toward COVID-19 vaccines. Responses are in a Likert format ranging from 0 = "Strongly Disagree") to 4 = "Strongly Agree"). Scores are tallied directly from 0 to 4, except for item 7, which is reverse-scored. Total scores range from 0 to 32, with higher scores indicating more favorable attitudes or acceptance of COVID-19 vaccines. The Spanish version demonstrated strong internal consistency (Cronbach's alpha of 0.94 and McDonald's omega of 0.95) and a unidimensional structure with acceptable goodness-of-fit indicators (CFI = 0.94, TLI = 0.91, SRMR = 0.04) (Campo-Arias et al., 2021).

### Procedure

Data collection for this study was limited to Phases 2 and 3. Phase 2 data were gathered between August and November 2021 through an online questionnaire. In contrast, Phase 3 data collection occurred from May to October 2022, utilizing both a self-administered pencil-and-paper questionnaire and an online format.

In both phases, participants received an explanation of the study and were asked to sign an informed consent form. This form outlined the research objectives, participants' rights, and the anonymity and confidentiality of their involvement. Participation was entirely voluntary, with no rewards or incentives offered. The mean response time to complete the questionnaires was between 15 and 20 min for both phases.

The Scientific Ethics Committee of the Universidad de Tarapacá granted ethical approval for this research, conducted as part of the FONDECYT Regular Project n°1,220,664.

### Data analysis

In Phase 2, parallel analysis was performed to establish the dimensionality of the instrument using the method of minimum residual estimation and oblimin rotation (Goretzko et al., 2021). To debug the scale, iterative debugging was performed based on three criteria: (1) retaining items with substantial factor loadings (λ > 0.5); (2) removing redundant items (Abad et al., 2011); and (3) removing items with large cross-loadings (> 0.3) (Muthén & Asparouhov, 2012; Xiao et al., 2019). An item analysis using the corrected homogeneity index followed, with indices greater than 0.05 deemed adequate and significant. This process resulted in a 14-item scale across two dimensions.

For Phase 3, an Exploratory Structural Equation Model (ESEM) with GEOMIN rotation (Asparouhouv & Muthén, 2009) and the weighted least squares estimation method were performed to establish evidence of validity based on the internal structure of the test. This estimation method is robust for non-normal discrete variables (DiStefano & Morgan, 2014; Li, 2016). Given the ordinal structure of the data, a polychoric correlation matrix was also used (Barendse et al., 2015). Reliability for each dimension was estimated using non-ordinal versions of Cronbach's alpha and McDonald's omega coefficients (Viladrich et al., 2017). Measurement invariance across different genders was assessed through multigroup (i.e., metric and scalar) confirmatory factor analysis (MG-CFA), considering Comparative Fit Index (CFI) decreases of less than 0.010 as evidence of invariance (Chen, 2007). Since a survey was applied in a pencil-and-paper and online format, invariance was also performed for the application format. Furthermore, evidence of validity based on the relationship with other variables was established through Set-ESEM (employing GEOMIN rotation, weighted least squares estimation method estimator, and polychoric correlations), examining the relationship between the dimensions of the naive skepticism scale and attitudes towards COVID-19 vaccines.

Model fit was evaluated following Schreiber’s (2017) recommended cut-point indicators: the CFI, the Tucker-Lewis Index (TLI), and the Root Mean Square Error of Approximation (RMSEA) (e.g., CFI > 0.95; TLI > 0.95; RMSEA < 0.06). Notably, parallel analysis, reliability coefficients, and the homogeneity index were obtained using Jamovi program v2.0.0 (The Jamovi Project, 2020), while the ESEM was conducted using Mplus v8.2 (Muthén and Muthén, 1998–2017).

## Results

### Phase 2: Pilot study

#### Parallel analysis

**Fig. 1:**
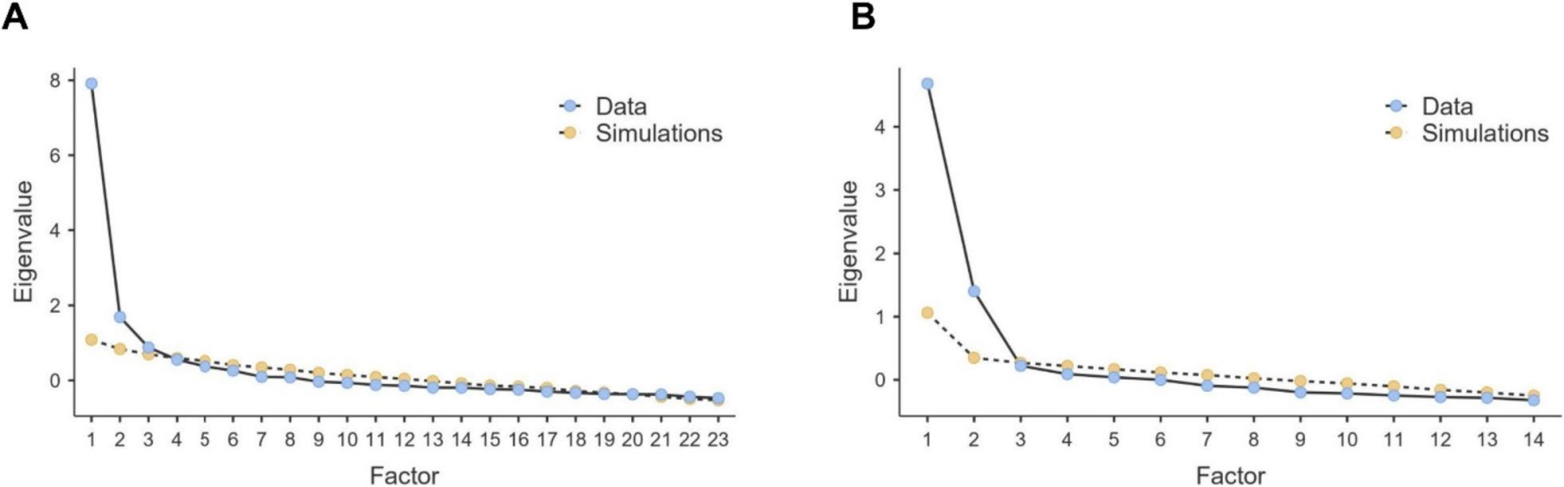
Parallel analysis model. **A** 23-item version; **B** 14-item version

In the 23-item version of the scale, parallel analysis suggested a three-factor solution (refer to Fig. 1A). The first 4 eigenvalues of this version were: 1 = 79.131; 2 = 16.878; 3 = 0.8765; 4 = 0.5506. However, upon closer examination of this structure, six items were identified with cross-loadings or saturations lower than 0.4. Consequently, an iterative revision and debugging process was undertaken, focusing on the content of the items and the corrected homogeneity index. This revision resulted in a debugged two-dimensional scale (see Fig. 1B) comprising 14 items: (a) SGO (7 items); and (b) SS (7 items) (illustrated in Fig. 1). The first 3 eigenvalues for this version were: 1 = 46.830; 2 = 14.015; 3 = 0.2222.

### Phase 3: Validity testing

#### Evidence of validity based on the internal structure of the test

The ESEM with the 14-item version of the NSS exhibited fit indicators aligning with literature recommendations (Schreiber, 2017). The model demonstrated satisfactory fit: χ2(64) = 190.597; CFI = 0.966; TLI = 0.951; RMSEA = 0.079 (confidence interval: 0.066—0.092); SRMSR = 0.034. Factor saturations and reliability estimates are presented in Table 2.

**Table 2:** Descriptive information of the NSS and resulting factor loadings from the ESEM

Naive Skepticism (NSS)	Mean (*SD*)	S	K	Factor^a^ loadings		α if the item is discarded	ω if the item is discarded
				A	B		
**Skepticism towards governmental organizations and official press (SGO)**							
** Los medios de prensa oficiales entregan información falsa** (The official media provide false information)	3.24 (0.75)	0.67	2.67	.681**	.003	.827	.829
** Desconfío de la información que entregan las autoridades gubernamentales** (I distrust the information provided by government authorities)	3.29 (0.93)	-0.19	-0.60	.711**	.001	.821	.824
** La Organización Mundial de la Salud (OMS) oculta sus verdaderos intereses** (The World Health Organization (WHO) hides its true interests)	2.89 (1.10)	1.71	-1.29	.637**	.272	.812	.815
** La prensa mundial manipula la información** (The world press manipulates information)	3.65 (0.96)	-2.79	-0.92	.791**	-.050	.808	.810
** Las redes sociales tildan de locos a quienes dicen verdades incómodas** (Social networks call those who tell uncomfortable truths crazy)	3.54 (0.96)	-1.55	-1.22	.538**	.079	.833	.835
** Los ricos manipulan la información de la prensa** (The rich manipulate press)	3.82 (1.02)	-2.57	-2.84	.758**	-.073	.820	.823
** Los organismos internacionales solo entregan la información que les beneficia** (International organizations only deliver information that benefits them)	3.38 (1.02)	-0.35	-1.80	.699**	.170	.808	.814
**Skepticism towards science (SS)**							
** Los artículos científicos mienten** (Scientific articles lie)	2.06 (0.85)	3.83	-0.66	.171	.724**	.816	.819
** Creo en las personas que desconfían de la ciencia** (I believe in people who distrust science)	2.13 (1.01)	3.45	-2.02	.068	.525**	.845	.848
** La ciencia aporta poco a la sociedad** (Science contributes little to society)	1.68 (1.08)	11.16	6.17	-.015	.779**	.823	.830
** Desconfío de las investigaciones científicas** (I distrust scientific research)	1.90 (0.94)	6.39	1.00	-.009	.840**	.811	.816
** Los científicos solo buscan obtener dinero** (Scientists are only out to make money)	2.40 (1.02)	2.26	-1.52	-.012	.717**	.824	.830
** Los hallazgos científicos son manipulados** (Scientific findings are manipulated)	2.53 (0.96)	2.53	-0.04	.256	.624**	.821	.827
** Los estudios que prueban los medicamentos son inútiles** (Studies that test medications are useless)	1.92 (0.98)	6.19	0.31	.158	.642**	.825	.831
				Correlation		Coefficient α	Coefficient ω
Skepticism towards governmental organizations and official press (SGO)	3.40 (0.69)	-.99	-.079	–		.841	.843
Skepticism of science (SC)	2.09 (0.70)	4.60	.078	.370**	–	.845	.850

*SD* Standard deviation, *S* skewness, *K* Kurtosis ** = *p* < .001; ^a^ = factor loadings of ESEM with two covariate factors, 14-item solution (M2)

Item factor saturations for each dimension indicated strong representation (SGO, λ = 0.53—0.79; SS, λ = 0.52—0.84) with low cross-factor saturation (SGO, λ = -0.07—0.27; SS, λ = -0.01—0.25). Reliability estimates were adequate for both SGO (α = 0.841, ω = 0.843; Cho & Kim, 2015) and SS (α = 0.845, ω = 0.850; Cho & Kim, 2015).

### Factorial invariance

The CFI deltas did not show a decrease in fit higher than 0.010 in either the metric or scalar models compared to the configuration model (i.e., multiple group CFA by sex). This finding suggests an equivalence in factor loadings and factor intercepts between male and female participants, indicating that the items hold consistent meaning across these groups. Regarding the application format, the CFI deltas also did not show a decrease in fit higher than 0.010 in in either the metric or scalar models compared to the configuration model. This suggests an equivalence in factor loadings and factor intercepts between the paper-and-pencil and online application formats. A t-test was also conducted to assess whether there were differences between the two application formats. The results of the t-test for independent samples showed statistically significant differences in the dimension skepticism towards governmental organizations and official press (Student's t(318) = 2.49; *p* = 0.013; Cohen's d = 0.337; online: M = 3.45, SD = 0.71; paper-and-pencil: M = 3.22, SD = 0.59) and skepticism towards science (Welch's t(318) = 2.52; *p* = 0.013; Cohen's d = 0.310; online: M = 2.13, SD = 0.73; paper-and-pencil: M = 1.93, SD = 0.53). In short, participants showed higher levels of skepticism towards government organizations and official press and skepticism towards science in the online application format compared to the pen and paper format. Detailed invariance tests conducted on sex and application format, using the final version of the scale are presented in Table 3.

**Table 3:** Fit indexes for multi-group confirmatory factor analysis of the NSS by sex and application format

	*χ*2	*df*	*χ*^2^/ *df*	RMSEA	90% CI	CFI	TLI	SRMR	CMs	Δ CFI
MG-CFA by Sex										
M1. Configural invariance	308.893	152	2.032	.081	[.068-.094]	.906	.887	.070	–	–
M2. Metric invariance	319.015	164	1.945	.077	[.065-.090]	.907	.897	.075	M2-M1	.001
M3. Scalar invariance	331.025	176	1.880	.075	[.062-.087]	.907	.904	.075	M3-M1	.001
MG-CFA by Application										
M4. Configural invariance	297.551	152	1.957	.077	[.064-.090]	.910	.892	.067	–	–
M5. Metric invariance	308.097	164	1.878	.074	[.061-.087]	.911	.901	.074	M5-M4	.001
M6. Scalar invariance	330.521	173	1.910	.074	[.062-.086]	.905	.901	.076	M6-M4	-.005

χ2 = *MG-CFA* Multi-group Confirmatory Factor Analysis; Chi-square, *df* Degree of freedom, *RMSEA* Root Mean Square Error of Approximation, *90% CI* Confidence Interval, *CFA* Confirmatory factor analysis, *CFI* Comparative Fit Index, *TLI* Tucker-Lewis Index, *SRMR* Standardized Root Mean Square Residual; CMs Comparisons between models; Δ CFI = CFI differential

### Evidence of validity based on the relationship with other variables

**Fig. 2:**
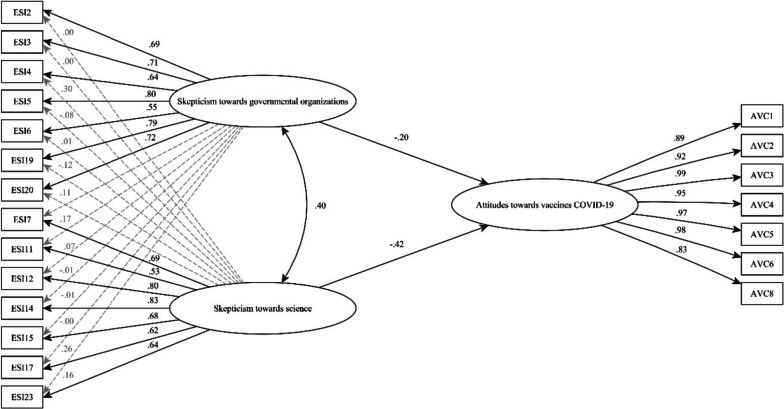
Set-ESEM model, graphical representation of the relationships between naive skepticism and attitudes towards vaccines COVID-19

The impact of the final version of the NSS on attitudes towards COVID-19 vaccines was evaluated using a Set-ESEM. This model demonstrated satisfactory fit indicators: χ2(174) = 354.029; CFI = 0.966; TLI = 0.955; RMSEA = 0.057 (confidence interval: 0.048—0.065); and Standardized Root Mean Square Residual = 0.039, thus aligning with recommendations by Schreiber (2017) (illustrated in Fig. 2).

The model revealed moderate, inverse, and statistically significant relationships between the latent variables; SGO and SS were inversely correlated with attitudes towards COVID-19 vaccines (γ = -0.202, *p* < 0.001 for SGO; γ = -0.425, *p* < 0.001 for SS, respectively).

## Discussion

The primary objectives of this study were to develop a scale for measuring naive skepticism among adults in Chile and to gather preliminary psychometric evidence to support its interpretation and application in researching risk factors associated with misinformation. The fit statistics of the 14-item model, the magnitude of factor loadings, and the lack of relevant cross-loadings substantiate the model's two-dimensional structure. These findings provide evidence of validity based on the internal structure, ensuring accurate interpretation of the scores. Furthermore, reliability coefficient estimates affirm that each dimension exhibits a satisfactory consistency level.

The 14-item model also demonstrated metric and scalar measurement invariance across genders, allowing the application of the scale to both men and women. This invariance signifies that the factor loadings were equivalent between groups, and the dimensions exhibited similar variability across sexes. Consequently, this new scale presents an opportunity to investigate the gender similarity hypothesis in susceptibility to misinformation in future research, as proposed by Hyde (2014). On the other hand, the model also demonstrated metric and scalar invariance between application formats, allowing for application in pencil and paper and online formats. It should be noted that the existence of slight differences in means between the application formats is not sufficient to suggest the use of one format over another. Furthermore, there is no differential functioning of the instrument (i.e., evidence of invariance). Therefore, for future psychometric testing, the application formats can be used in combination or separately.

In terms of validity evidence based on the association with other variables, the dimensions of the NSS were found to correlate with attitudes toward COVID-19 vaccines, aligning with the anticipated direction and corroborating prior studies (Bavel et al., 2020; Roozenbeek & van der Linden, 2019; Roozenbeek et al., 2020). These studies have indicated that negative attitudes towards vaccines are frequently underpinned by mistaken beliefs about the consequences of such behaviors, stemming from either ignorance or a predisposition to accept false or biased information. This inclination, potentially influenced by individual factors like naive skepticism, increases vulnerability to misinformation (Wright, 2020). Consequently, this misinformation hampers the development of behaviors essential for health prevention and maintenance, inadvertently encouraging behaviors that compromise health.

### Limitations and implications

The main limitation of this study lies in the size and representativeness of the sample. Being non-probabilistic, the generalizability of the findings to the broader population is constrained. Accordingly, it is recommended that future psychometric studies utilizing this instrument be extended to diverse groups, such as adolescents, older adults, and individuals from varied socioeconomic backgrounds and educational levels, as well as in the medical, health, and educational contexts.

Given the nascent stage of this field, future research should also explore the convergent and discriminant validity of the NSS in comparison with scales measuring beliefs in conspiracy theories (e.g., Generic Conspiracist Beliefs Scale, Brotherton et al., 2013; Belief in Conspiracy Theories Inventory, Swami et al., 2010) and critical thinking (e.g., The Critical Thinking Disposition Scale, Sosu, 2013). This approach will enable the differentiation and correlation of these theoretical constructs.

Considering the significant impact of naive skepticism on health behaviors (Wang et al., 2019; Zheng et al., 2022), incorporating this new scale into assessment protocols within health services or educational centers could prove beneficial. The insights garnered from this instrument could aid in identifying individuals susceptible to misinformation and the practice of risky behaviors, thereby necessitating tailored preventive interventions. Consequently, current strategies promoting health risk behaviors in adults could be enhanced, focusing on the adoption of certain behaviors and the avoidance of others.

## Conclusion

The final 14-item version of the NSS demonstrated evidence of reliability and validity. This evidence, grounded in the internal structure of the test, measurement invariance, and associations with other variables, supports the applicability of the scale in sample groups akin to those in this study. The preliminary findings indicate that this scale represents a novel, concise instrument crafted using modern psychometric techniques. The NSS provides an updated and alternative proposal to assess naive skepticism and holds potential for use in researching psychological factors associated with health risk behaviors.

## Data Availability

The datasets used and/or analyzed during the current study are available from the corresponding author on reasonable request.
